# An improved kinetic model for the acetone-butanol-ethanol pathway of *Clostridium acetobutylicum* and model-based perturbation analysis

**DOI:** 10.1186/1752-0509-5-S1-S12

**Published:** 2011-06-20

**Authors:** Ru-Dong Li, Yuan-Yuan Li, Ling-Yi Lu, Cong Ren, Yi-Xue Li, Lei Liu

**Affiliations:** 1 Key Laboratory of Systems Biology, Shanghai Institutes for Biological Sciences (SIBS), Chinese Academy of Sciences (CAS), Shanghai, China; 2 Shanghai Center for Bioinformatics Technology (SCBIT), Shanghai, China; 3 Institute of Plant Physiology and Ecology, Shanghai Institutes for Biological Sciences (SIBS), Chinese Academy of Sciences (CAS), Shanghai, China

## Abstract

**Background:**

Comprehensive kinetic models of microbial metabolism can enhance the understanding of system dynamics and regulatory mechanisms, which is helpful in optimizing microbial production of industrial chemicals. *Clostridium acetobutylicum* produces solvents (acetone-butanol–ethanol, ABE) through the ABE pathway. To systematically assess the potential of increased production of solvents, kinetic modeling has been applied to analyze the dynamics of this pathway and make predictive simulations. Up to date, only one kinetic model for *C. acetobutylicum* supported by experiment has been reported as far as we know. But this model did not integrate the metabolic regulatory effects of transcriptional control and other complex factors. It also left out the information of some key intermediates (e.g. butyryl-phosphate).

**Results:**

We have developed an improved kinetic model featured with the incorporation of butyryl-phosphate, inclusion of net effects of complex metabolic regulations, and quantification of endogenous enzyme activity variations caused by these regulations. The simulation results of our model are more consistent with published experimental data than the previous model, especially in terms of reflecting the kinetics of butyryl-phosphate and butyrate. Through parameter perturbation analysis, it was found that butyrate kinase has large and positive influence on butanol production while CoA transferase has negative effect on butanol production, suggesting that butyrate kinase has more efficiency in converting butyrate to butanol than CoA transferase.

**Conclusions:**

Our improved kinetic model of the ABE process has more capacity in approaching real circumstances, providing much more insight in the regulatory mechanisms and potential key points for optimization of solvent productions. Moreover, the modeling strategy can be extended to other biological processes.

## Background

**Figure 1:**
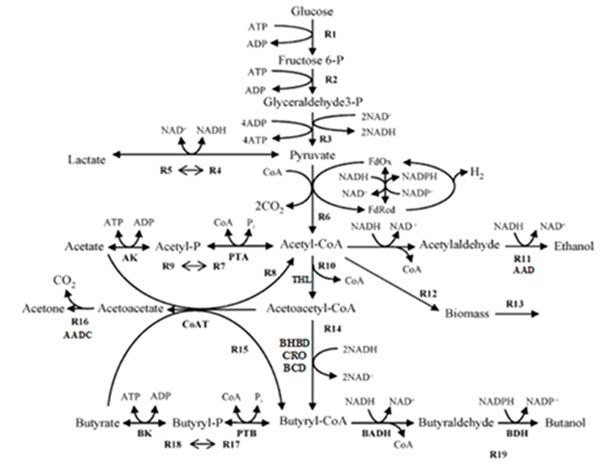
**The acetone-butanol-ethanol (ABE) pathway of *C. acetobutylicum*.** Reactions are represented by bold arrows and denoted by symbols from R1 to R21. The acidogenic reactions are R9 and R18 (catalyzed by PTA-AK and PTB-BK, respectively), generating acetate and butyrate respectively. The two acids are reassimilated through R7 and R17 (the reverse paths of R9 and R18), or directly converted to acetyl-CoA and butyryl-CoA through R8 and R15 (catalyzed by CoAT). The solventogenic reactions are R11, R16 and R19 (catalyzed by AAD, AADC and BDH, respectively), generating ethanol, acetate and butanol respectively. And R14 is a lumped reaction consisted of reactions catalyzed by BHBD, CRO and BCD [14]

System modeling for metabolism of industrial microorganisms is important in metabolic engineering, as a comprehensive model can reveal relevant factors related to high yield of target products. Based on such analyses, system modeling can further enhance developing operation strategies, or help optimizing cultivation processes [1-4]. *C. acetobutylicum* is an extensively studied organism used for industrial-scale production of important solvents acetone and butanol, through the acetone-butanol-ethanol (ABE) pathway (Figure 1) [5,6]. The ABE pathway of *C. acetobutylicum* comprises two distinct branches: acidogenesis and solventogenesis. During acidogenesis, cells grow exponentially, acetate and butyrate are vigorously produced and the solvents (butanol, acetone and ethanol) are not obviously generated. While shifting to solventogenesis, the cells arrest their growth at stationary phase, solvents are produced and acids are reassimilated [5].

So far, multiple models have been established to simulate the ABE pathway, which mostly apply the metabolic flux analysis (MFA) and flux-balance analysis (FBA) approaches [5,7-10]. Although stoichiometric models can simulate the overall flux distributions based on limited kinetic data by using physicochemical constraints, they cannot appropriately reflect the dynamics in real-time scale. In contrast, kinetics models integrated with biochemical information are more efficient in reflecting system dynamics. By perturbing a kinetic model, system states that deviate from the normal state can be simulated and it is possible to reveal which reactions have potential impacts on target products’ productions. To date, many experiments have explored the kinetic features of the ABE process of *C. acetobutylicum*[11-13] and a kinetic model was recently developed by Shinto* et al*. [14]. However, as most current models did, this model did not integrate the metabolic regulatory effects of transcriptional control and other complex factors [15-17]. Moreover, Shinto’s model did not include the information of some key metabolites, e.g. butyryl-phoshate (BuP), which has proved to be important in solventogensis [16-19].

To overcome the drawbacks of Shinto’s model, we developed an improved kinetic model for *C. acetobutylicum* ABE process. The simulation results based on our model were consistent with published experimental observations and more comprehensive than those of Shinto’s model. Furthermore, a series of perturbed circumstances were simulated as well, getting results which might provide insights for metabolic engineering aiming at increasing solvent productivity.

## Results

All the following results were based on our new model (Equation (1), section “Methods”), and they were compared with an experimental study (Zhao *et al*., 2005) that was independent of Shinto’s model or our work. The new model was established by integrating experimental information and knowledge not included in Shinto’s model (section “Methods”), and we applied some optimization methods to fix the unknown parameters introduced by integrating these information and knowledge. The parameter fitting was done only under the conditions described in Shinto’s work, only with respect to the metabolites contained in Shinto’s model, and we didn’t use any information related to BuP or the experimental study for comparison (Zhao *et al*., 2005). After these parameter values were derived, we first implemented dynamic simulation with respect to the conditions in Zhao *et al.*’s work and compared the results with experimental observations. We then carried out perturbation analysis to detect which reactions had large impacts on the overall butanol production in the system.

### Dynamical simulation

**Figure 2:**
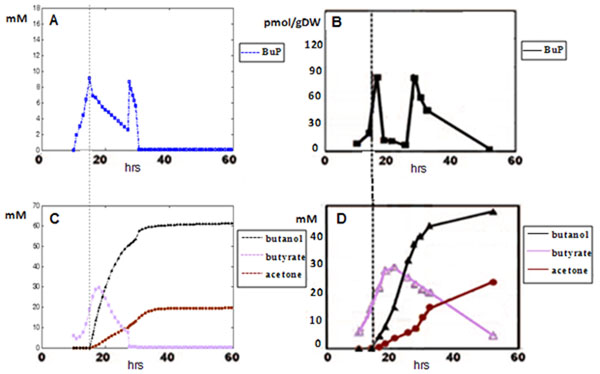
**Comparison of simulation results with the experimental observations of Zhao *et al.*** A is the simulation results of BuP kinetics, in which data spots are concentrations and represented in blue. B is the experimental observation of BuP kinetics, in which data are concentrations and represented in black [16]. C is the simulation results of the kinetics of butanol (black), butyrate (light pink) and acetone (brown). D is the experimental observations of the kinetics of butanol (black), butyrate (light pink) and acetone (brown) [16]. In A, C and D, the metric unit of vertical axis is mM; in B, it is pmol/gDW. In A, B, C and D the unit of lateral axis is hr. The figure shows that the simulated curves are consistent with the experimental ones, both in quantity scale and shape.

The initial value of our model was set according to the conditions described in the experiment by Zhao *et al.* (2005) [16], and the simulation results of metabolites’ kinetic profiles were shown in Figure 2A and 2C. Since our model parameters were fitted under Shinto’s experiment conditions, the metabolites’ kinetic profiles were naturally consistent with those in Shinto’s experiment when Shinto’s conditions were substituted in. So the comparison with Shinto’s experiment was not shown and we only focused on comparing with Zhao *et al.*’s experiment here. These simulation results were shown to be quite consistent with experimental observations (Figure 2B and 2D). The metric units in Figure 2A and 2B were different (Figure 2A: mM; Figure 2B: pmol/gDW), since the measurement of BuP in Zhao *et al*’s experiment accepted the unit of pmol/gDW. It was impossible to exactly know the conversion between mM and pmol/gDW because there was no such relationship established in the SI metric unit system. But the quantity scale could be approximated given the size of an ordinary *C. acetobutylicum* cell, and this scale was consistent with that in our simulation results. Since bacteria cells might vary in their sizes, we could not give a general estimation that could represent all the others, so we just showed the original quantities on the vertical axis in Figure 2B.

**Figure 3:**
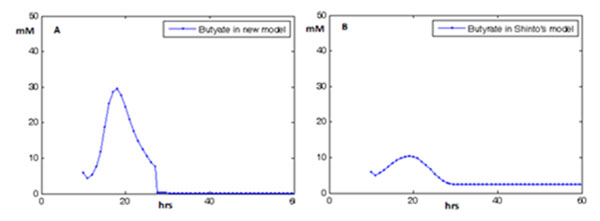
**Comparison of simualtion results of butyrate kinetics with Shinto’s model under Zhao *et al*’s conditions** A is the simulation results of butyrate kinetics based on our new model under Zhao *et al.*’s conditions. B is the simulation results of butyrate kinetics based on Shinto’s model under Zhao *et al.*’s conditions. The data spots are concentrations. The metric unit of the vertical axis in A and B is mM; and the unit of lateral axis is hr. In A and B, A is more consistent with the observation of butyrate in Zhao *et al.*’s experiment (Figure 2D). The figure shows that our simulated curve of butyrate kinetics is more accurate than the one produced by Shinto’s model, both in quantity scale and shape.

In our simulation results, the first peak of BuP was shown to coincide with the onset of solvent production (Figure 2A, 2C). This was a phenomenon that was reported in experimental literatures and had biological implications [16-19]. Besides BuP, we also demonstrated that we had a more precise simulation on butyrate, one crucial product in cell growth and solvent production [5] (Figure 3). In Shinto’s model, when substituting in Zhao *et al.*’s conditions, the quantity scale of butyrate curve (Figure 3B) didn’t resemble precisely with the experimental curve in Zhao *et al.*’s experiment (Figure 2D). This further demonstrated that our model had more capability in approaching real biological events.

### Perturbation analysis

Among the solvents (ethanol, acetone, butanol) produced in the ABE fermentation, butanol was considered to be the more valuable product, since it had advantageous properties over acetone and ethanol (e.g. better value for the heat of combustion) [6]. So we implemented a series of perturbation analyses to assess which enzymes/reactions had relatively large impacts on butanol production. Here we used *Rd* values to measure the impacts (see section “Methods” for *Rd*’s definition). We carried out perturbation tests both on single parameters and double parameter pairs, the magnitude was 5% and shift directions (upward/downward) were considered. We traversed the entire parameter set. The result set of single parameter perturbations included  entries (additional file 1 and 2). And the result set of double parameter perturbations included  entries (additional file 3, 4, 5, 6). Here Table 1 and 2 showed some results with respect to the enzymes located on or close to acidogenic/solventogenic reactions in single and double parameter perturbations, respectively.

**Table 1:** Part of the results of single parameter perturbation analysis.

P +5%			P -5%		
P	Enz	Rd	P	Enz	Rd

Vmax19	BDH	0.0076	Vmax19	BDH	-0.0082

Vmax17	BK	0.0061	Vmax17	BK	-0.0063

Vmax18	PTB	-0.006	Vmax18	PTB	0.0063

Vmax14	B-C-B	0.0076	Vmax14	B-C-B	-0.0082

Vmax11	AAD	-0.0003	Vmax11	AAD	0.0003

Vmax7	AK	0.0054	Vmax7	AK	-0.0054

Vmax9	PTA	-0.0012	Vmax9	PTA	0.0012

Vmax15	CoAT^a^	-0.0072	Vmax15	CoAT	0.0074

Vmax8	CoAT^b^	-0.0002	Vmax8	CoAT	0.0002

Vmax1	PTS	0.0088	Vmax1	PTS	-0.0088

This table lists part of the results of perturbation tests on single kinetic parameters. Here “P” denotes the parameter perturbed. “Enz” denotes the corresponding enzyme. “+/- 5%” indicates up/down-shifting the parameter value by 5%. “B-C-B” stands for enzyme series BHBD-CRO-BCD. Subscripts “a” and “b” indicate there are 2 CoA transferases catalyzing R8 and R15 and here we took them in uniform.

**Table 2:** Part of the results of double parameter perturbation analysis

P1+5%, P2+5%					P1-5%, P2-5%				
P1	Enz1	P2	Enz2	Rd	P1	Enz1	P2	Enz2	Rd

Vmax14	B-C-B	Vmax19	BDH	0.0153	Vmax14	B-C-B	Vmax19	BDH	-0.0163

Vmax14	B-C-B	Vmax17	BK	0.0138	Vmax14	B-C-B	Vmax17	BK	-0.0145

Vmax15	CoAT	Vmax17	BK	-0.001	Vmax15	CoAT	Vmax17	BK	0.001

Vmax17	BK	Vmax19	BDH	0.0137	Vmax17	BK	Vmax19	BDH	-0.0146

Vmax18	PTB	Vmax19	BDH	0.0016	Vmax18	PTB	Vmax19	BDH	-0.0018

Vmax7	AK	Vmax8	CoAT	0.0053	Vmax7	AK	Vmax8	CoAT	-0.0053

Vmax9	PTA	Vmax11	AAD	-0.0014	Vmax9	PTA	Vmax11	AAD	0.0014

Vmax1	PTS	Vmax14	B-C-B	0.0163	Vmax1	PTS	Vmax14	B-C-B	-0.0171

Vmax1	PTS	Vmax19	BDH	0.0163	Vmax1	PTS	Vmax19	BDH	-0.0172

P1+5%, P2-5%					P1-5%, P2+5%				

P1	Enz1	P2	Enz2	Rd	P1	Enz1	P2	Enz2	Rd

Vmax14	B-C-B	Vmax19	BDH	-0.0007	Vmax14	B-C-B	Vmax19	BDH	-0.0007

Vmax14	B-C-B	Vmax17	BK	0.0012	Vmax14	B-C-B	Vmax17	BK	-0.002

Vmax15	CoAT	Vmax17	BK	-0.0135	Vmax15	CoAT	Vmax17	BK	0.0136

Vmax17	BK	Vmax19	BDH	-0.0021	Vmax17	BK	Vmax19	BDH	0.0012

Vmax18	PTB	Vmax19	BDH	-0.0143	Vmax18	PTB	Vmax19	BDH	0.0138

Vmax7	AK	Vmax8	CoAT	0.0057	Vmax7	AK	Vmax8	CoAT	-0.0056

Vmax9	PTA	Vmax11	AAD	-0.0009	Vmax9	PTA	Vmax11	AAD	0.0009

Vmax1	PTS	Vmax14	B-C-B	0.0008	Vmax1	PTS	Vmax14	B-C-B	-0.0012

Vmax1	PTS	Vmax19	BDH	0.0008	Vmax1	PTS	Vmax19	BDH	-0.0011

This table lists part of the results of perturbation tests on double parameter pairs. Here “P1” and “P2” denote the parameters perturbed. “Enz1” and “Enz2” denote the corresponding enzymes. “+/- 5%” and “B-C-B” stand for the same meaning as in Table 1.

Among all results, there were several interesting ones that might provide some insights for understanding the ABE process. Before examining the results, we could intuitively hypothesize that BK might be relatively important in solventogensis since it connected two important metabolites butyrate and BuP. Based on the analyses, we indeed found that shifting BK’s *V_max_* alone or in combination with other enzyme parameters (e.g. the apparent *V_max_* parameter of BHBD – CRO – BCD) resulted in relatively large influences on butanol production (Table 1, 2). Actually, BK activity had positive effect on butanol production and the change in butanol quantity caused by shifting BK’s *V_max_* ranked the 5th in the profile of single parameter shifts (see Table 1 and additional file 1, 2). This suggested that BK, which coupled PTB to generate butyrate as well as catalyzing butyrate reassimilation, was important to butanol production as compared with other enzymes such as AAD (indexed as R11). Besides, AK also had positive effect on butanol production (but with a smaller *Rd* values than BK, see Table 1), indicating acetate reassimilation had similar influence as butyrate reassimilation in solventogenesis, but with less magnitude.

**Figure 4:**
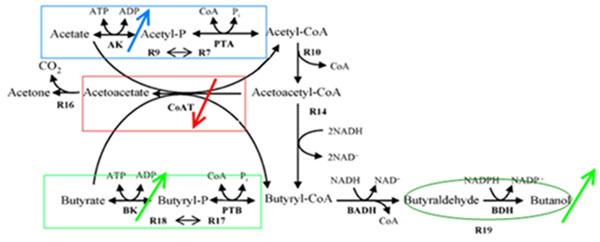
**Illustration of the influences on butanol production originated from BK, CoAT and AK.** The influence originated from BK is positive effect on butanol production, as indicated by the green arrow in the same direction with that of butanol production. The influence originated from CoAT is negative effect on butanol production, as indicated by the red arrow in the opposite direction with that of butanol production. The influence originated from AK is also positive effect on butanol production, but with a smaller magnitude than that of BK. It’s indicated by the blue arrow in the same direction with that of butanol production.

Also, our computation results showed that CoAT, which also accepted butyrate as substrate, had a negative effect on butanol production as up-shifting its catalytic capacity (increasing *V_max_* or decreasing *K_m_*) diminished butanol quantity (Table 1, 2). Because up-shifting the catalytic capacity of BK (increasing its *V_max_* or decreasing its *K_m_*) or down-shifting the catalytic capacity of CoAT (decreasing its *V_max_* or increasing its *K_m_*) would cause more butyrate molecules received by BK and the reverse operations would cause more butyrate received by CoAT, and given the fact that BK and CoAT both accepted butyrate as substrate, we could see that if more butyrate was received by BK, the butanol production would increase, i.e. more butyrate molecules were converted and acid reassimilation was more efficient; and if more butyrate was received by CoAT, the situation would be on the contrary (Figure 4). Therefore we could conclude that BK had more efficiency than CoAT during acid reassimilation and solventogenesis.

There were some places where our new model’s predictions differed from those of Shinto’s model. For instance, our model predicted that PTS had positive influence on butanol production, as increasing its *V_max_* (or decreasing its *K_m_*) resulted in amplified *Rd* value. While by Shinto’s model, PTS’s influence was negative. Given the fact that PTS acted in nutrient uptake and many processes relating to the ABE pathway were subjected to nutrient induction, our prediction might be more intuitively consistent with common sense [20].

## Discussion

Rational system modeling and comprehensive system analysis can serve as prior guidelines for understanding and deducing biological mechanisms. We can retrieve quantitative knowledge for assessing an organism’s metabolic capacity and use this knowledge for in-lab experiments to develop new strains with advantageous productivity [3,4], or optimizing the cultivation process of existing strains [2].

### Model improvements

Since many studies related to *C. acetobutylicum* ABE pathway have been reported (including parameter values and the rate equation formulas [14,21]), kinetic modeling of the ABE pathway becomes feasible and enables us to simulate the system dynamics. Nevertheless, the previous kinetic model of *C. acetobutylicum* ABE process (Shinto *et al*, 2007) has several drawbacks as described in earlier context. To overcome these drawbacks, we have established a new model featured with three improvements over the previous one.

First, we have incorporated the key metabolite BuP, reflecting the relevant biological events that are specific to ABE kinetics [15-18]. The correspondence between BuP concentration climax and solventogenesis onset is not merely a natural consequence of the fact that BuP is the intermediate between butyryl-CoA and butyrate. There are implications on the genetic level as stated in Zhao *et al*’s study [16-19]. There are many important solventogenic genes, such as *adh*E1 (CAP0162), *adh*E2 (CAP0035 ), *ctf*A (CAP0163), *ctf*B (CAP0164), *adc* (CAP0165), *bdh*A (CAC3298), *bdh*B (CAC3299), etc., having expression profiles that show a strictly correlated pattern with the kinetics of BuP. Although the detailed mechanism of how BuP acts to regulate ABE process has not been very clear yet, its functional importance has been experimentally confirmed [16-19]. Our new model accounts for this knowledge and is successful in representing the phenomenon.

Second, we describe the regulatory effects of complex factors using a time division pattern. In Shinto’s model, the metabolic regulation beyond the level of substrate/product inhibition/activation is simply defined as the input of glucose. The shut-downs of several acidogenic/solventogenic enzymes (like PTB, BDH, etc) are solely due to the insufficiency of glucose. However, various evidences indicate that even with sufficient supply of glucose, the acidogenic enzymes are still shut down in the solventogenic phase, and the solventogenic enzymes are necessarily inactivated at the beginning of the acidogenic phase [15,22,23]. Therefore, the metabolic regulations are not of the simple pattern as Shinto suggested, but a significant 2-phase mode is shown (acids are generated during the earlier phase and solvents are generated during the latter one). In our work, this mode is approximated by considering endogenous enzyme activity variations, assuming enzymes are regulated by many factors (e.g. transcription control) to exhibit different activity levels to fulfill conditional system requirements of different periods. This assumption is equivalent to extending the application of biochemical system theory (BST). In BST, which is based on *in vitro* experiments, enzyme concentrations and endogenous enzyme activity levels are constant by default. Hence kinetic models based on BST are rigorously suitable for chemical simulations but may not entirely appropriate for *in vivo* conditions. Under *in vivo* conditions, the rate of a reaction does not solely depend on substrate/product concentrations, because the endogenous enzyme activity itself is regulated by many factors and its variation in turn affects the reaction rate [15-17]. Our model divides time into a set of periods according to the enzymes’ activity variations, allowing enzyme activities to vary throughout these periods.

Third, we introduce the “enzyme activity coefficient (EAC)” to quantify endogenous enzyme activity variations caused by metabolic regulations (see section “Methods” for EAC’s definition). For the quantification of enzyme activity curves, numerical interpolation (e.g. Lagrange, Legendre, etc.) should have been employed as to obtain fully continuous functions. But measurements in activity assays are usually not precise. If the errors are large, interpolation may result in huge errors or mistakes, causing the trouble of overfitting and distorting the original curve profile. On the contrary, the computation of EAC leaves the error just as the original error. Hence, using EAC will at least not amplify the error or distort the curve when the measurements are not precise. Moreover, our design of EAC is calculating a ratio instead of the particular value at each time instance, and this allows the error to be divided by a denominator, thus lowering the error level in computation.

### Dynamical simulation and perturbation analysis

After the addition of BuP, 5 unknown parameters are introduced into the system. We have used Genetic Algorithm to estimate their values. In the process of parameter estimation, we used Shinto’s experimental observations of 16 metabolites to formulate the fitness function, but we didn’t employ any information about BuP. And in order to avoid the mistake of reasoning in a circle, we compare our results with observations of another experiment (Zhao *et al.*’s). It turns out that our results are significantly consistent with the observations and have shown some superiority over Shinto’s model in reflecting the kinetics of BuP and butyrate. This indicates that Shinto’s model is well fitted for its own condition but may not be suited well for other conditions. In contrast, our model has more capacity in approaching real cases because of the improvements we have made.

Simulations based on kinetic models can help develop in-lab strategies, thus increasing the success rate of metabolic engineering. In our work, we have simulated thousands of perturbed conditions to detect and assess potential spots that have large influences on butanol production. The magnitude of *in silico* perturbations should not be too large because the system may exhibit alternative activations for other pathways when undergoing substantial fluctuations [24,25]. When the system is encountering slight perturbations, its overall properties will not change substantially due to biological robustness [25-27]. So it would be fairly assumed that when the perturbation magnitude on enzymatic parameters is 5%, the system will still survive and its functional normality is not interrupted or diverted. In the computation, we have identified an interesting phenomenon that BK’s catalytic capacity exhibits positive influence on butanol production while CoAT has negative influences, as elevating BK activity results in increased *Rd* value and uplifting CoAT activity diminishes the value. And more convincingly, *Rd* decreases when increasing the *V_max_* values of BK and CoAT at the same time, which means the negative effect of CoAT can balance the positive effect of BK, confirming that CoAT has large effect in impairing butanol production. Based on this discovery, we propose a possible scenario that if more metabolites are received by BK as substrates, the overall acid (butyrate) reassimilation efficiency will be benefited and butanol production is enhanced. And if more metabolites are received by CoAT as substrates, the situation will be on the contrary. It may not seem economical for the bacteria to use the BK-PTB path (see Figure 1) to reassimilate butyrate since running through this path consumes ATP. Nonetheless, based on our computation results and biochemical knowledge, we raise a predictive explanation for the underlying mechanism: in acidogenic phase, the metabolic flux actually runs in the direction of PTB-BK (confirmed by both our computation of kinetic profiles and experimental literature [5,16,21]), thus this path generates ATP for the growth of the bacteria; when the bacteria enters solventogenic phase, it doesn’t need to grow and ATP has surplus, these surplus ATPs are utilized to proceed butyrate reassimilation. It’s noteworthy that acids are severely poisonous to bacteria cells and it is a priority for the bacteria to convert acids to other forms (e.g. alcohol). In addition, enhanced butanol production means more acids are converted. Hence, although reactions through BK cost ATP, but so far as BK’s efficiency is concerned, BK is still the preferred enzyme through which the bacteria reassimilates butyrate during solventogenesis. Therefore, the reason why the ATP-costing path BK-PTB is more efficient over the path catalyzed by CoAT (not ATP-costing) in reassimilating butyrate is probably because of responding to severe poison stress, and the energetic basis for this process is the ATP surplus generated during acidogenic phase. Our prediction is equivalent to considering the bacterial cellular behaviour to be related with biological robustness, as supposing that the bacteria is not seeking for its optimality when undergoing stress response, but seeking for sub-optimality. In such case, certain costs or sacrifices are tolerated as long as it can survive (or maintain minimal fluctuation from normality) [24,26].

In double parameter perturbation tests, we noticed that the net effect of combinatorial perturbation was equal to the sum of effects of individual perturbations, indicating that no crossover or nonlinear amplification originated from perturbations with mild magnitudes. This is probably because when the system is undergoing mild perturbation, it tries to maintain the normal status with minor alterations by means of system robustness. To demonstrate the hypothesis further, we implemented some three-parameter combinatorial perturbation tests. We randomly chose a number of three-parameter triplets and randomly decided their shift directions. For example, if we increased three parameters *V*_max14_, *V*_max19_, *V*_max17_ by 5% each and re-computed our model (Equation (1)), we obtained *Rd*=2.14%, which exactly equalled the sum of individual effects of these perturbations. Again, if we increased *K*_*m*15*b*_ and *V*_max19_ by 5% and decreased *V*_max18_ by 5%, we obtained *Rd*=2.09%, still equalled to the sum of individual effects. Hence, we raise a hypothetic measure for increased butanol production: By slightly perturbing parameters in suitable directions and with appropriately mild magnitudes, we possibly can obtain a metabolic phenotype that can have amplified butanol production, and the strain can steadily and safely survive as well. The amplification magnitudes of multiple parameter perturbations can be much greater than those in single parameter perturbations, if adequately many parameters are manipulated appropriately. Meanwhile, from an engineering point of view, multiple spot modifications can make the risks of system fluctuations or external impulsions more distributive than in the case that all alterations are concentrated on a single spot. Hence, this strategy provides a way that can make a more stable high-production system. But this strategy requires high-precision genetic manipulation.

### Significance

Traditional kinetic models cannot accommodate complex metabolic regulation effects (e.g. gene transcriptional control). Hence previous integrative modeling approaches for metabolic system are mainly based on the FBA method, in which the gene transcription regulations are described by Boolean logic and the metabolic level is expressed by flux balance equations. Since FBA based methods and Boolean logic cannot adequately reflect system dynamics, we have developed a new model as an attempt towards solving the problem. Actually, our modeling strategy is equivalent to extending the traditional BST, degenerating complex metabolic regulation effects to a form that is compatible with kinetic models. This strategy provides a way for integrating complex factors and knowledge from multiple levels into the framework of kinetic models. Moreover, our approach of describing metabolic regulation effects with a time division pattern and EAC is extendable. For instance, we can relate the enzyme activities to gene transcriptional level, build a formulism between them, and include the effects of other factors such as impulse and stochasticity. Our modeling method can be generalized and extended to the modeling of other bio-processes.

In this post-genomic era, massive information and experimental data have been accumulated. Therefore, it is important to develop methods or tools that are able to make use of existed information/data and capable of organizing, manipulating and interpreting them more comprehensively [28,29]. Our work just attempts to serve that goal by integrating existed information from multiple aspects and describing them mathematically. Nevertheless, the usage of “net effects of regulatory factors” in our modeling doesn’t seem to build direct links between the genetic level and metabolic level. But if adequately more information about the regulatory factors on the genetic level is revealed, better formulism can be built to link the two levels and further studies on the control of bacteria cellular systems can be conducted.

## Conclusions

We have developed a new kinetic model featured with major improvements over the previous one (Shinto’s model), with the information of BuP incorporated and the effects of complex metabolic regulatory factors included. The simulation results based on our model are highly consistent with published experimental data and have more superiority in precision and subtlety than the previous model. We have successfully simulated the right profile of BuP kinetics, which is not included in the previous model. And we can make more precise prediction on the kinetics of butyrate, another important intermediate in the ABE process. Through perturbation analysis, we predict that the path catalyzed by BK is more efficient over the one catalyzed by CoAT in converting butyrate to butanol during solventogenesis, although ATPs are consumed.

## Methods

We made improvements to Shinto’s model with respect to three points: (i) incorporating key compound butyryl-phosphate (BuP); (ii) describing the net effects of complex ABE metabolic regulations with a time division pattern according to endogenous enzyme activity variations, and (iii) introducing the “enzyme activity coefficient” to quantify endogenous enzyme activity variations. After the model framework was established, parameter estimation was followed to obtain unknown parameter values. We then implemented perturbation analysis to detect sensitivities of reactions/enzymes.

### Incorporating BuP

BuP was key intermediate in conversions between butyrate (But) and butyryl-CoA (BCoA). It was reported that BuP played a crucial role in solventogenesis, as the initial peak of its concentration marked the onset of solvent production [16]. Adding in BuP meant splitting the originally lumped reactions between But and BCoA (as in Shinto’s model) so as to represent their intermediate BuP as a system component. Here we added two new reactions to denote the conversions from BuP to But and BCoA respectively. Hence, the butyrate formation/reassimilation branch was restructured and BuP appeared as another system component. Mathematically, we created rate equations for the new reactions and re-formulated the mass balance equations relating to But, BCoA and BuP. For details, see additional file 8.

### Time division pattern

We assumed endogenous enzyme activity variations were net effects of transcriptional control and other complex factors. As experimental studies suggested enzyme activities varied with time [15,21-23,30], we developed a time division pattern to reflect the regulatory effects. We divided time into several intervals according to the enzymes’ activity variation profiles [22,23]. Here we only considered a subset of enzymes, which were either located on acid/solvent production reactions or directly associated to them. We adopted activity variations of the enzymes in consideration and regarded others’ as constants. All enzyme activity profiles were collected from published experimental studies [22,23] and the experiments were done under the identical culture conditions as our simulation [14,16]. For details of constructing the time division pattern, see additional file 8.

### Enzyme activity coefficient

We introduced EAC to quantify endogenous enzyme activity variations. EACs were formulated as time-dependent functions. At each time instance, the EAC value was the ratio of the current enzyme activity to its maximum activity. Here we employed the divided intervals in the time division pattern (see the previous paragraph) as markers of time. And for computation simplicity, we approximated EAC with a set of 0^th^ splines with respect to these markers. In other word, the EAC value remained constant within a divided interval and changed to another constant when stepping into another interval. The constant was the ratio of the average activity level in the interval to the maximum activity. We calculated all EACs of the considered enzymes and multiplied them to their corresponding rate equations to reflect endogenous activity variations. All enzyme activities data were collected from literatures [22,23]. For details of computing EAC, see additional file 8.

### New Model

The new model contained 21 rate equations and 17 differential equations, involving 50 kinetic parameters. The model was built by integrating ABE kinetic features identified so far. Except for those included in Shinto’s model [11-14], EACs were multiplied to rate equations. The model was expressed in the form of ordinary differential equation (ODE) system as in Equation (1):(1)

where ***Y*** was the vector of metabolites’ concentrations; ***A*** was the stoichiometric matrix of mass balance equations; ***E***=diag{EAC_1_,…,EAC_21_} and EACs corresponding to enzymes with constant activities were set to 1;  was the vector of rate equations without EACs; and ***P*** was the entire set of parameters. For details of the equations, symbols and abbreviations in the model, see additional file 8.

### Unknown parameter estimation

We applied Genetic Algorithm (GA) to *de novo* estimate unknown parameters introduced by new reactions (previous subsection “Incorporating BuP”). We considered the experimental observations of 16 metabolites in Shinto’s work to be valid, and assumed that the correct value assignment of the unknown parameters definitely reproduced these valid observations under Shinto’s conditions. Therefore the fitness function in optimization was formed by forcing the 16 metabolites’ concentrations ***Y***(1:16) to match Shinto’s observations ***Y_0_***(1:16). We computed parameter values that minimized the fitness function and accepted them as numerical solutions. In addition, we didn’t employ any qualitative or quantitative information of BuP or Zhao *et al*.’s experiment in this process. For parameter values, see additional file 7. And for details of parameter estimation, see additional file 8.

### Perturbation analysis

We performed perturbation analysis to assess enzymes/reactions’ impacts on butanol production. By consecutively shifting the enzymes’ *V_max_* and *K_m_* values and using the normal state as control, relative changes of *in silico* butanol production were computed. We defined the relative change in butanol production as *Rd* (a ratio expressed in Formula (2)):(2)

where ***y_p_*** was the instantaneous butanol concentration in perturbed state, and ***y_c_*** was that in normal state. For approximation, we discretized the integrals in Formula (2) with the trapezoid method. The results of perturbation analysis were in additional files 1, 2, 3, 4, 5, 6, and for details of computation, see additional file 8.

## List of abbreviations

PTS: phosphotransferase system; AK: acetate kinase; PTA: phosphotransacetylase; CoAT: CoA transferase; AAD: alcohol/ aldehyde dehydrogenase; BHBD: β-hydroxybutyryl-CoA dehydrogenase; CRO: crotonase; BK: butyrate kinase; PTB: phosphotransbutyrylase; BDH: butanol dehydrogenase; BCD: butyryl-CoA dehydrogenase; AADC: acetoacetate decarboxylase; THL: thiolase

## Competing interests

The authors declare that they have no competing interests.

## Authors' contributions

Building the kinetic model: RDL and CR. Designing and performing numerical experiments: RDL and LYL. Data acquisition and analysis: RDL. Conceiving and designing the research: YYL, LL, YXL. Drafting the manuscript: RDL, YYL, LL.

## Supplementary Material

Additional file 1 **Results of single parameter perturbation tests with magnitude +5%.** The data entries included are numerical results obtained by increasing the value of every kinetic parameter by 5%. All parameters are traversed. There are 50 entries and the dataset is organized as a table in the format of *.xls (Excel worksheet). The first column is the index of the parameter perturbed, the second column is the parameter perturbed, and the third column is the *Rd* value that is used to evaluate how much impact the perturbation causes to butanol production. Click here for file

Additional file 2 **Results of single parameter perturbation tests with magnitude -5%.** The data entries included are numerical results obtained by decreasing the value of every kinetic parameter by 5%. All parameters are traversed. There are 50 entries and the dataset is organized as a table in the format of *.xls (Excel worksheet). The first column is the index, the second column is the parameter perturbed, and the third column is the *Rd* value that is used to evaluate how much impact the perturbation causes to butanol production. Click here for file

Additional file 3 **Results of double parameter perturbation tests with respective magnitudes +5% and +5%.** The data entries included are numerical results obtained by increasing the values of every pair of kinetic parameters by 5% each. All 2-parameter combinations are traversed. There are 1225 entries and the dataset is organized as a table in the format of *.xls (Excel worksheet). The first column is the pair of indexes of the parameters perturbed, the second and third columns are the parameters perturbed, respectively, and the fourth column is the *Rd* value that is used to evaluate how much impact the perturbation causes to butanol production. Click here for file

Additional file 4 **Results of double parameter perturbation tests with respective magnitudes +5% and -5%.** The data entries included are numerical results obtained by altering the values of every pair of kinetic parameters, increasing the first parameter by 5% and decreasing the other one by 5%. All 2-parameter combinations are traversed. There are 1225 entries and the dataset is organized as a table in the format of *.xls (Excel worksheet). The first column is the pair of indexes of the parameters perturbed, the second and third columns are the parameters perturbed, respectively, and the fourth column is the *Rd* value that is used to evaluate how much impact the perturbation causes to butanol production. Click here for file

Additional file 5 **Results of double parameter perturbation tests with respective magnitudes -5% and +5%.** The data entries included are numerical results obtained by altering the values of every pair of kinetic parameters, decreasing the first parameter by 5% and increasing the other one by 5%. All 2-parameter combinations are traversed. There are 1225 entries and the dataset is organized as a table in the format of *.xls (Excel worksheet). The first column is the pair of indexes of the parameters perturbed, the second and third columns are the parameters perturbed, respectively, and the fourth column is the *Rd* value that is used to evaluate how much impact the perturbation causes to butanol production. Click here for file

Additional file 6 **Results of double parameter perturbation tests with respective magnitudes -5% and -5%.** The data entries included are numerical results obtained by decreasing the values of every pair of kinetic parameters by 5% each. All 2-parameter combinations are traversed. There are 1225 entries and the dataset is organized as a table in the format of *.xls (Excel worksheet). The first column is the pair of indexes of the parameters perturbed, the second and third columns are the parameters perturbed, respectively, and the fourth column is the *Rd* value that is used to evaluate how much impact the perturbation causes to butanol production. Click here for file

Additional file 7 **The values of kinetic parameters** There are 50 parameters in our kinetic model. The dataset is organized as a table in the format of *.xls (Excel worksheet). The first column contains the indexes of reactions, the second column contains the parameters involved in each reaction, and the third column contains the parameter values. Click here for file

Additional file 8 **Description of the modeling method** This is the detailed description of the method of modeling, including the incorporation of BuP, the construction of time division pattern, the computation of EACs, parameter estimation procedure, and the computation of perturbation analysis. This file is in the format of * (Word document). This file contains 4 supplementary figures (Figure S1 - S4) and a supplementary table (Table S1). Click here for file
