# Supplementary material for: An improved kinetic model for the acetone-butanol-ethanol pathway of Clostridium acetobutylicum and model-based perturbation analysis
Source: BMC Syst Biol. 2011 Jun 20;5(Suppl 1):S12. doi: 10.1186/1752-0509-5-S1-S12 (PMC3121112; doi:10.1186/1752-0509-5-S1-S12)
Supplement: Additional file 8 — Description of the modeling method This is the detailed description of the method of modeling, including the incorporation of BuP, the construction of time division pattern, the computation of EACs, parameter estimation procedure, and the computation of perturbation analysis. This file is in the format of * (Word document). This file contains 4 supplementary figures (Figure S1 - S4) and a supplementary table (Table S1). [file 1752-0509-5-S1-S12-S8.doc]

# Additional file 8

## S1. Incorporating butyryl-phosphate

Butyryl-phosphate (BuP) is the intermediate of the conversion between butyrate (But) and butyryl-CoA (BCoA) in acidogensis and acid reassimilation (R17-R18 in Figure 1). It is reported that butyryl-phosphate plays a crucial role in solvent production, as the initial peak of its concentration marks the onset of solvent production [1]. Adding BuP means splitting the originally lumped reactions R17 and R18 (Figure 1), so as to represent their intermediate BuP as a system component. Here we use R17 to denote the conversion from butyrate (But) to BuP and R20 to denote the subsequent conversion from BuP to butyryl-CoA (BCoA). Likewise, the reverse process is also decomposed into two steps, with R18 denoting the reversal of R20 and R21 denoting the reversal of R17. Hence, the butyrate formation branch is restructured and BuP appears as another component in the system (Figure S1).

Similar to the rate equation formulations in Shinto’s work, R20 and R21 are assumed to be of the following Michaelis-Menten forms,

$r_{20}=\frac{V_{\max20}[BuP][Biom]}{K_{m20}\left(1+K_{a20}/[But]\right)+[BuP]}$ (1)

$r_{21}=\frac{V_{\max21}[BuP][Biom]}{K_{m21}+[BuP]}$ (2)

where $V_{\max20},V_{\max21},K_{m20},K_{m21},K_{a20}$are kinetic parameters. In $r_{20}$, a term representing the activation effect in the denominator is added based on the conclusion raised by Tashiro *et al.* [2] that the presence of butyrate can enhance the reaction with activation parameter$K_{a20}$.

Aftermath, 2 differential equations relating to BCoA and But must be updated to include $r_{20},r_{21}$according to Figure S1, as the following:

$\frac{d[BCoA]}{dt}=r_{14}+r_{15}+r_{20}-r_{18}-r_{19}$ (3)

$\frac{d[But]}{dt}=r_{21}-r_{15}-r_{17}$ (4)

And 1 new differential equation relating to BuP must be created, as the following:

$\frac{d[BuP]}{dt}=r_{17}+r_{18}-r_{20}-r_{21}$ (5)

## S2. Time division pattern

We assume that the endogenous enzymes’ activity variations are net effects of transcriptional control and other complex factors. For instance, when a gene is up-regulated, its corresponding enzyme will have elevated concentration and apparently increased activity. But an enzyme’s activity is not proportional to the transcription of its corresponding gene, as other complex factors are involved and many of these factors remained unrevealed. So only “enzyme activity” can serve as a parameter that inclusively reflect the effects mediated by these factors involved in metabolic regulation.

We develop a time division pattern to reflect metabolic regulatory effects in acidogenesis/solventogenesis, as experimental studies suggest enzyme activities varied with time [3-7]. We divide time according to the enzymes’ activity variation profiles [5, 6]. Here we only consider a subset of all the enzymes. Members of this subset are either located right on the acid/solvent production reactions or directly associate to them. This subset includes: AK, CoAT, PTA, THL, AAD, BHBD, CRO, AADC, BCD, BK, PTB, and BDH [3-7]. We adopt the activity variations of these enzymes, while the activities of the other enzymes are regarded as constants. We consider reactions catalyzed by BHBD, CRO and BCD to be a lumped reaction because these enzymes’ activity variations have a very similar pattern [5, 8].

For example, consider the two activity curves of PTA and THL in Figure S2. In the PTA curve, the PTA activity in interval (0, 5hr) is vigorous, and its activity in (5hr, 17hr) is also vigorous but lower than that in (0, 5hr). After 17hr, PTA activity is very low. Hence, we can divide the time axis according to this pattern as:

$\sum_{PTA}=(0,5)\cup(5,17)\cup(17,+\infty)$ (6)

For the THL curve, a different pattern is shown. In (0, 5hr), THL activity is at a median level, in (5hr, 17 hr), its activity is vigorous, in (17hr, 19.5hr), THL activity undergoes a short transition, and after 19.5hr, the activity goes down to a status that was lower than the initial median level. So a division of time according THL activity is:

$\sum_{THL}=(0,5)\cup(5,17)\cup(17,19.5)\cup(19.5,+\infty)$ (7)

We can then summarize divisions and as the intersection of them. This is assumed to be the common time division according to both PTA and THL. For every enzyme mentioned above, there is a division. And after all these divisions are calculated, we can calculate the intersection of all of them as the overall time division, which is suitable for the timing of all the enzymes’ activity variation profiles. So we can write the overall time division ***T*** as

$T=\sum_{AK}\cap\sum_{CoAT}\cap\cdots\cap\sum_{BDH}$ (8)

where every subscript is an enzyme mentioned here. We have derived the ***T*** of our model in this way and summarized the biological features the enzymes exhibited in each divided interval of ***T*** (Table S1). For convenience of illustration, ***T*** is depicted in Figure S3, in which the divided intervals are denoted from T1 to T5 and drawn in proportion to their sizes. Acidogenesis actually contained T1 and T2, and solventogenesis contained T3, T4 and T5. Noteworthy, for convenience of computation, the time in ***T*** is model time, which is 10hr later than the real time. If compared with real time-course observations, a calibration of 10hr has to be made (+10hr to the time scale of ***T***). This overall division ***T*** is used throughout our model as a reflection of metabolic regulation. In different time intervals, the *in vivo* conditions for enzymes are different, and enzymes are regulated to exhibit different endogenous activity levels to fulfill conditional system requirements [3, 9]. All enzyme activity profiles were collected from published experimental works [5, 6], and the referred experiments were done under the identical culture conditions as our simulation [1, 10].

## S3. Enzyme activity coefficient

We introduce “enzyme activity coefficient” (EAC) to quantify endogenous enzyme activity variations. EACs are formulated as time-dependent functions. At each time instance, EAC is defined as the ratio of the current enzyme activity to its maximum activity. And here we employ the divided intervals in overall division ***T*** (see the previous paragraph, Formula (8)) as markers on the time axis. For simplicity, we approximate the EAC with a set of 0^th^ splines. In other word, the EAC value remains constant within every divided interval in ***T***; when stepping into another interval, EAC value changes to another constant. The constant in each interval represents the average activity level (divided by the enzyme’s maximum activity) in this interval base on experimental measurements from literatures [5, 6]. For example, consider PTA and AK again, their experimental enzyme activity curves are shown in Figure S2A. According to overall time division ***T***, the first interval is (0, 5hr). We calculate the average level of PTA activity in this interval and divide it by PTA’s maximum activity, thus we obtained the EAC value in (0, 5hr). And in the other intervals of ***T***, the same calculations are carried out, obtaining corresponding EAC values and finally the EAC function of PTA is formed. The EAC function of AK can be formed in the same way, and these curves of EAC functions are shown in Figure S4. Note that in intervals (17 hr, 19.5hr), (19.5hr, 28.5hr) and (28.5hr, +∞), the enzyme activities of PTA (AK) are the same, therefore there is one EAC value after time point 17hr. The piece-wise EAC functions of AK and PTA were described in Formula (9) and (10).

$EAC_{AK}(t)=\left\{\begin{matrix} 1,t\in[0,5] \\ 1/6,t\in(5,17] \\ 0.08,t\in(17,+\infty) \end{matrix}\right.$ (9)

$EAC_{PTA}(t)=\left\{\begin{matrix} 1,t\in[0,5] \\ 5/6,t\in(5,17] \\ 0.05,t\in(17,+\infty) \end{matrix}\right.$ (10)

By its nature, EAC is a coefficient with no metric unit and no more than 1.

For the consistency with reactions, we index the EACs with the indices of their corresponding reactions (see Figure 1, and amended by Figure S1), instead of the enzyme names. There are 13 EACs (indexed by 7~11 and 14~21) in total and they are shown by Formula (11) – (23) as follows. All EAC values are calculated from published experimental literatures[5, 6] .

$EAC_{7}\left(t\right)=\left\{\begin{matrix} 1,t\in[0,5] \\ 1/6,t\in(5,17] \\ 0.08,t\in(17,+\infty) \end{matrix}\right.$ (11)

$EAC_{8}\left(t\right)=\left\{\begin{matrix} 0.1,t\in[0,5] \\ 1,t\in(5,+\infty) \end{matrix}\right.$ (12)

$EAC_{9}\left(t\right)=\left\{\begin{matrix} 1,t\in[0,5] \\ 5/6,t\in(5,17] \\ 0.05,t\in(17,+\infty) \end{matrix}\right.$ (13)

$EAC_{10}\left(t\right)=\left\{\begin{matrix} 4/7,t\in[0,5] \\ 1,t\in(5,17] \\ 6/7,t\in(17,19.5] \\ 37/70,t\in(19.5,+\infty) \end{matrix}\right.$ (14)

$EAC_{11}\left(t\right)=\left\{\begin{matrix} 0,t\in[0,5] \\ 1,t\in(5,+\infty) \end{matrix}\right.$ (15)

$EAC_{14}\left(t\right)=\left\{\begin{matrix} 9/14,t\in[0,5] \\ 1,t\in(5,17] \\ 5/14,t\in(17,19.5] \\ 1/7,t\in(19.5,+\infty) \end{matrix}\right.$ (16)

$EAC_{15}\left(t\right)=\left\{\begin{matrix} 0.1,t\in[0,5] \\ 1,t\in(5,+\infty) \end{matrix}\right.$ (17)

$EAC_{16}\left(t\right)=\left\{\begin{matrix} \begin{matrix} 0,t\in[0,5] \\ 0.005,t\in(5,17] \\ 0.01,t\in(17,19.5] \\ 1/60,t\in(19.5,28.5] \end{matrix} \\ 1,t\in(28.5,+\infty) \end{matrix}\right.$ (18)

$EAC_{17}\left(t\right)=\left\{\begin{matrix} 31/175,t\in[0,5] \\ 53/350,t\in(5,17] \\ 1,t\in(17,28.5] \\ 0,t\in(28.5,+\infty) \end{matrix}\right.$ (19)

$EAC_{18}\left(t\right)=\left\{\begin{matrix} 1,t\in[0,5] \\ 0.328,t\in(5,17] \\ 0,t\in(17,+\infty) \end{matrix}\right.$ (20)

$EAC_{19}\left(t\right)=\left\{\begin{matrix} 0,t\in[0,5] \\ 1,t\in(5,+\infty) \end{matrix}\right.$ (21)

$EAC_{20}(t)=\left\{\begin{matrix} 1,t\in[0,5] \\ 0.328,t\in(5,17] \\ 0.031,t\in(17,19.5] \\ 1,t\in(19.5,28.5] \\ 0,t\in(28.5,+\infty) \end{matrix}\right.$ (22)

$EAC_{21}(t)=\left\{\begin{matrix} 1,t\in[0,17] \\ 0.06,t\in(17,19.5] \\ 1,t\in(19.5,28.5] \\ 0,t\in(28.5,+\infty) \end{matrix}\right.$ (23)

The EACs are multiplied with their corresponding enzymes’ rate equations to reflect enzyme activity variations. And here again, the time variable is the model time, a calibration of +10hr has to be made when compared with real time observations, as mentioned earlier.

## S4. New Model

The new kinetic model is consisted of 21 rate equations and 17 differential equations, involving 21 reactions, 17 metabolites and 50 kinetic parameters. The model is built by integrating the kinetic features of ABE pathway identified so far. Except for the biochemistry knowledge included in Shinto’s model [2, 10-12], EACs are multiplied with corresponding reactions to reflect the net effects of complex metabolic regulatory factors. See section “List of abbreviations” for the descriptions of symbols of metabolites and parameters.

The rate equations are listed as follows

$r_{1}=\frac{V_{max1}[Glu][Biom]}{\left\{Km\left(1+[Glu]/K_{is1}\right)+[Glu]\right\}\left(1+[Bul]/K_{ii1}\right)}$ (24)

$r_{2}=\frac{V_{\max2}[F6P][Biom]}{K_{m2}+[F6P]}$ (25)

$r_{3}=\frac{V_{\max3}[G3P][Biom]}{K_{m3}+[G3P]}$ (26)

$r_{4}=\frac{V_{\max4}[Lac][Biom]}{K_{m4}+[Lac]}$ (27)

$r_{5}=\frac{V_{\max5}[Pyr][Biom]}{K_{m5}+[Pyr]}$ (28)

$r_{6}=\frac{V_{\max6}[Pyr][Biom]}{K_{m6}+[Pyr]}$ (29)

$r_{7}=\frac{V_{\max7}[Act][Biom]}{K_{m7}+[Act]}\cdot EAC_{7}(t)$ (30)

$r_{8}=V_{\max8}\left(\frac{1}{1+K_{m8a}/[Act]}\right)\left(\frac{1}{1+K_{m8b}/[AACoA]}\right)[Biom]\cdot EAC_{8}(t)$ (31)

$r_{9}=\frac{V_{\max9}[ACoA][Biom]}{K_{m9}+[ACoA]}\cdot EAC_{9}(t)$ (32)

$r_{10}=\frac{V_{\max10}[ACoA][Biom]}{K_{m10}+[ACoA]}\cdot EAC_{10}(t)$ (33)

$r_{11}=\frac{V_{\max11}[ACoA][Biom]}{K_{m11}+[Biom]}\cdot EAC_{11}(t)$ (34)

$r_{12}=\frac{V_{\max12}[ACoA][Biom]}{K_{m12}\left(1+[But]/K_{ii12}\right)+[ACoA]\left(1+[Bul]/K_{ii12}\right)}$ (35)

$r_{13}=k_{13}[Biom]$ (36)

$r_{14}=\frac{V_{\max14}[AACoA][Biom]}{K_{m14}+[AACoA]}\cdot EAC_{14}(t)$ (37)

$r_{15}=V_{\max15}\left(\frac{1}{1+K_{m15a}/[But]}\right)\left(\frac{1}{1+K_{m15b}/[AACoA]}\right)[Biom]\cdot EAC_{15}(t)$ (38)

$r_{16}=\frac{V_{\max16}[AcAct][Biom]}{K_{m16}+[AcAct]}\cdot EAC_{16}(t)$ (39)

$r_{17}=\frac{V_{\max17}[But][Biom]}{K_{m17}\left(1+K_{a17}/[But]\right)+[But]}\cdot EAC_{17}(t)$ (40)

$r_{18}=\frac{V_{\max18}[BCoA][Biom]}{K_{m18}+[BCoA]}\cdot EAC_{18}(t)$ (41)

$r_{19}=\frac{V_{\max19}[BCoA][Biom]}{\left\{K_{m19}\left(1+K_{a19}/[But]\right)+[BCoA]\right\}\left(1+[Bul]/K_{ii19}\right)}\cdot EAC_{19}(t)$ (42)

$r_{20}=\frac{V_{\max20}[BuP][Biom]}{K_{m20}\left(1+K_{a20}/[But]\right)+[BuP]}\cdot EAC_{20}(t)$ (43)

$\text{r}_{21}=\frac{V_{\max21}[BuP][Biom]}{K_{m21}+[BuP]}\cdot EAC_{21}(t)$ (44)

In these equations, [*Biom*] represents the concentration of biomass, which can be expressed by formula $\text{CH}_{p}\text{O}_{n}\text{N}_{q}$[13]. The content ratio of C, H, O and N (i.e. the exact values of p, n, q) can be measured and the average molecular weight of biomass can be obtained (here it is set to be 172 as in Shinto’s work). The kinetic parameters in Equation (24) - (44) are actually apparent kinetic parameters that are suitable for the formulism of these equations [10].

The differential equations representing the mass balance of metabolites are shown as follows:

$\frac{d[Glu]}{dt}=-r_{1}$ (45)

$\frac{d[F6P]}{dt}=r_{1}-r_{2}$ (46)

$\frac{d[G3P]}{dt}=r_{2}-r_{3}$ (47)

$\frac{d[Pyr]}{dt}=r_{3}+r_{4}-r_{5}-r_{6}$ (48)

$\frac{d[Lac]}{dt}=r_{5}-r_{4}$ (49)

$\frac{d[ACoA]}{dt}=r_{6}+r_{7}+r_{8}-r_{9}-r_{10}-r_{11}-r_{12}$ (50)

$\frac{d[Biom]}{dt}=r_{12}-r_{13}$ (51)

$\frac{d[Act]}{dt}=r_{9}-r_{7}-r_{8}$ (52)

$\frac{d[Etl]}{dt}=r_{11}$ (53)

$\frac{d[AACoA]}{dt}=r_{10}-r_{8}-r_{14}-r_{15}$ (54)

$\frac{d[AcAct]}{dt}=r_{8}+r_{15}-r_{16}$ (55)

$\frac{d[BCoA]}{dt}=r_{14}+r_{15}+r_{20}-r_{18}-r_{19}$ (56)

$\frac{d[But]}{dt}=r_{21}-r_{15}-r_{17}$ (57)

$\frac{d[Acn]}{dt}=r_{16}$ (58)

$\frac{d[CO_{2}]}{dt}=r_{6}+r_{16}$ (59)

$\frac{d[Bul]}{dt}=r_{19}$ (60)

$\frac{d[BuP]}{dt}=r_{17}+r_{18}-r_{20}-r_{21}$ (61)

We can denote all metabolite concentrations as a column vector$\mathbf{Y}=\left([Glu],[F6P],...,[BuP]\right)^{T}$. Then we arrange the stoichiometric coefficients in differential equations (45) - (61) in a matrix$\mathbf{A}$(17×21 dimension). We then assigne $EAC_{i}\left(t\right)=1$for i= 1, 2, 3, 4, 5, 6, 12, 13 and collocate all the EACs in a diagonal matrix$\mathbf{E}(t)=diag\left(eac_{1},eac_{2},...,eac_{21}\right)$. We denote the j-th rate equation without being multiplied by EAC as $\tilde{r}_{j}$ ($r_{j}=\tilde{r}_{j}\cdot EAC_{j}$,$j=1,2,...,21$) and group all the $\tilde{r}_{j}$ in a 21-dimension column vector$\tilde{\mathbf{R}}=\left(\tilde{r}_{1},\tilde{r}_{2},...,\tilde{r}_{21}\right)^{T}$. In addition, we use symbol$\mathbf{P}$ to denote the set of kinetic parameters (or the vector comprises the collocation of all the kinetic parameters). Therefore, the new model can be expressed in the matrix form as in Equation (62),

$\frac{d\mathbf{Y}}{dt}=\mathbf{A}\cdot\mathbf{E}(t)\cdot\tilde{\mathbf{R}}(\mathbf{Y},\mathbf{P})$ (62)

Combining with an assigned initial value$\vec{Y}_{0}$, a Cauchy problem is formed by Equation (62) and simulations can be implemented.

## S5. Unknown parameter estimation

The apparent kinetic parameters with respect to reaction R20 and R21: $V_{\max20}$,$V_{\max21}$,$K_{m20}$, $K_{m21}$and $K_{a20}$are unknown, as there are no records of their exact values. Besides, since the parameters are apparent parameters, different kinetic equation formulism will generate different parameter values. So if some of them do have records of values but are not obtained according to the equation formulism in this work, we can not use them either.

Therefore we apply the Genetic Algorithm (GA) to *de novo* estimate these 5 unknown parameters. But we do not employ any information of BuP, neither qualitative nor quantitative, in the process of parameter estimation. In Shinto’s work, there are experimental observations for the concentrations of 16 metabolites, which are also present in our model (the first 16 metabolites, occur on the left sides of Equation (45) – (61)). We consider these observations to be valid under Shinto’s experiment condition, and use these 16 metabolites’ quantities for parameter estimation. We assume that the right value assignment of the 5 parameters will definitely reproduce the valid observations of these 16 metabolites under Shinto’s experiment condition. Hence, our strategy is forcing these 16 metabolites’ concentrations $\mathbf{Y}_{1:16}$to match the observation in Shinto’s experiment $\mathbf{Y}_{1:16}^{0}$. So the fitness function in our implementation of GA is:

$\min f(\mathbf{Y}_{1:16})=\left\|\mathbf{Y}_{1:16}-\mathbf{Y}_{1:16}^{0}\right\|_{2}$ (63)

And this optimization problem is defined in mathematics as the following:

$\begin{matrix} \min f(\mathbf{Y}_{1:16})=\left\|\mathbf{Y}_{1:16}-\mathbf{Y}_{1:16}^{0}\right\|_{2} \\ s.t.\left\{\begin{matrix} \frac{d\mathbf{Y}}{dt}=\mathbf{A}\cdot\mathbf{E}(t)\cdot\tilde{\mathbf{R}}(\mathbf{Y},\mathbf{P}) \\ \mathbf{Y}=\left([Glu],[F6P],...,[Bul],[BuP]\right)^{T}\in R^{17} \\ \mathbf{Y}_{1:16}=(\mathbf{Y}(1),\mathbf{Y}(2),...\mathbf{Y}(16))^{T}=([Glu],[F6P],...,[Bul])^{T}\in R^{16} \end{matrix}\right. \end{matrix}$ (64)

We fix all the known parameters in **P**, and let the 5 unknown ones to vary. By solving optimization problem (63) with GA, we have finally found out the 5 values that minimized the fitness function. And we accept these values as the numerical approximations of the unknown parameters. There are various factors that can influence the output and convergence of the algorithm (e.g. initial population size, generation number, fitness function variation limit, etc.). Therefore, adequately plenty of numerical experiments are required to be implemented. For simplicity, the detailed procedures are omitted here. The entire set of parameter values, including estimated unknown parameters values and all the other parameter values, are shown in additional file 7.

## S6. Perturbation analysis

We employ the idea of perturbation analysis to assess which enzymes/reactions have relatively large impacts on butanol production. We accomplish this by consecutively shifting the values of *V_max_* and *K_m_* in all the enzymes. The magnitude of perturbation on each enzyme’s parameters is assigned to be 5% and the relative increase or decrease of *in silico* butanol production is observed, using the un-perturbed (normal) state as control. In this way, the enzyme’s sensitivity to perturbation is assessed. We define the ratio of relative butanol production change as *Rd* (Formula (65)):

$Rd=\frac{\int_{t0}^{tf} y_{p}(t)dt-\int_{t0}^{tf} y_{c}(t)dt}{\int_{t0}^{tf} y_{c}(t)dt}$ (65)

where$y_{p}(t)$is the instantaneous butanol concentration of the perturbed state at time instance t, and$y_{c}(t)$is the un-perturbed one. The overall butanol productions in the respective states are expressed by integrating the two functions over time interval [t0, tf], which corresponds to the time span between the initial and end time points in simulation. As for approximation, we discretize the integrals on the right side of Formula (65) with the trapezoid method (Formula (66))

$\int_{T} f(t)dt=\sum_{k=0}^{N-1} \frac{f(t_{k})+f(t_{k+1})}{2}\cdot\left(t_{k+1}-t_{k}\right)$ (66)

where $T=\bigcup_{k=0}^{N-1} \left[t_{k},t_{k+1}\right]$ (67)

Therefore, *Rd* can be converted to be Formula (68):

$Rd\approx\frac{\sum_{k=0}^{N-1} \left(\frac{y_{p}(t_{k})+y_{p}(t_{k+1})}{2}\right)\left(t_{k+1}-t_{k}\right)-\sum_{k=0}^{N-1} \left(\frac{y_{c}(\tilde{t}_{k})+y_{c}(\tilde{t}_{k+1})}{2}\right)\left(\tilde{t}_{k+1}-\tilde{t}_{k}\right)}{\sum_{k=0}^{N-1} \left(\frac{y_{c}(\tilde{t}_{k})+y_{c}(\tilde{t}_{k+1})}{2}\right)\left(\tilde{t}_{k+1}-\tilde{t}_{k}\right)}$ (68)

If we take uniform time steps equal to unit length, i.e.$\forall t_{k}=\tilde{t}_{k},t_{k+1}-t_{k}=1$, a simple form can be obtained for the approximation of *Rd* (Formula (69)):

$Rd\approx\frac{\sum_{k=0}^{N-1} \left[\left(y_{p}(t_{k})+y_{p}(t_{k+1})\right)-\left(y_{c}(t_{k})+y_{c}(t_{k+1})\right)\right]}{\sum_{k=0}^{N-1} \left(y_{c}(t_{k})+y_{c}(t_{k+1})\right)}$ (69) where in our simulation, $t_{0}=0,t_{1}=1,...$and so forth.

List of abbreviations

| **Abbr** | **Description** |
| --- | --- |
| Glu | glucose |
| F6P | fructose-6-phosphate |
| G3P | glyceraldehydes-3-phosphate |
| Lac | lactate |
| Pyr | pyruvate |
| Act | acetate |
| AACoA | acetoacetyl-CoA |
| ACoA | acetyl-CoA |
| But | butyrate |
| Bul | butanol |
| AcAct | acetoacetate |
| BCoA | butyryl-CoA |
| BuP | butyryl-phosphate |
| CO2 | carbon dioxide or carbonate |
| Biom | biomass |
| PTS | phosphotransferase system |
| AK | acetate kinase |
| PTA | phosphotransacetylase |
| CoAT | CoA transferase |
| THL | thiolase |
| AAD | alcohol/ aldehyde dehydrogenase |
| BHBD | β-hydroxybutyryl-CoA dehydrogenase |
| CRO | crotonase |
| AADC | acetoacetate decarboxylase |
| BK | butyrate kinase |
| PTB | phosphotransbutyrylase |
| BDH | butanol dehydrogenase |
| BCD | butyryl-CoA dehydrogenase |
| *V_maxj_* | maximum reaction rate (h^-1^) in reaction j |
| *K_mj_* | Michaelis-Menten constant (mM) in reaction j |
| *K_aj_* | activation constant (mM) for activators in reaction j |
| *K_iij_* | inhibition constant (mM) for inhibitors in reaction j |
| *K_isj_* | inhibition constant (mM) for substrates in reaction j |
| *k_j_*  *r_j_* | conversion rate constant (h^-1^) for reaction j  rate equation of reaction j |
| R_j_ | metabolic reaction j in the ABE pathway |
| EAC*_j_* | the enzyme activity coefficient in reaction j |

**References**

1. Zhao Y, Tomas CA, Rudolph FB, Papoutsakis ET, Bennett GN: **Intracellular butyryl phosphate and acetyl phosphate concentrations in Clostridium acetobutylicum and their implications for solvent formation**. *Appl Environ Microbiol* 2005, **71**:530-537.

2. Tashiro Y, Takeda K, Kobayashi G, Sonomoto K, Ishizaki A, *et al*: **High butanol production by Clostridium saccharoperbutylacetonicum N1-4 in fed-batch culture with pH-stat continuous butyric acid and glucose feeding method**. *J Biosci Bioeng* 2004, **98**:263-268.

3. Alsaker KV, Papoutsakis ET: **Transcriptional program of early sporulation and stationary-phase events in Clostridium acetobutylicum**. *J Bacteriol* 2005, **187**:7103-7118.

4. Gheshlaghi R, Scharer JM, Moo-Young M, Chou CP: **Metabolic pathways of clostridia for producing butanol**. *Biotechnol Adv* 2009, **27**:764-781.

5. Hartmanis MGN, Gatenbeck S: **Intermediary metabolism in Clostridium acetobutylicum: Levels of enzymes involved in the formation of acetate and butyrate**. *Appl Environ Microbiol* 1984, **47**:1277-1283.

6. Tummala SB, Welker NE, Papoutsakis ET: **Development and characterization of a gene expression reporter system for Clostridium acetobutylicum ATCC 824**. *Appl Environ Microbiol* 1999, **65**:3793-3799.

7. Tomas CA, Beamish J, Papoutsakis ET: **Transcriptional analysis of butanol stress and tolerance in Clostridium acetobutylicum**. *J Bacteriol* 2004, **186**:2006-2018.

8. Nölling J, Breton G, Omelchenko MV, Makarova KS, Zeng Q, *et al*: **Genome Sequence and Comparative Analysis of the Solvent-Producing Bacterium *Clostridium acetobutylicum***. *J Bacteriol* 2001, **183**(16):4823-4838.

9. Thormann K, Feustel L, Lorenz K, Nakotte S, Durre P: **Control of butanol formation in Clostridium acetobutylicum by transcriptional activation**. *J Bacteriol* 2002, **184**:1966-1973.

10. Shinto H, Tashiro Y, Yamashita M, Kobayashi G, Sekiguchi T, *et al*: **Kinetic modeling and sensitivity analysis of acetone-butanol-ethanol production**. *J Biotechnol* 2007, **131**:45-56.

11. Jones DT, Woods DR: **Acetone-butanol fermentation revisited**. *Microbiol Rev* 1986, **50**(484-524).

12. Soni BK, Das K, Ghose TK: **Inhibitory factors involved in acetone-butanol fermentation by Clostridium saccharoperbutylacetonicum**. *Curr Microbiol* 1987, **16**:61-67.

13. Papoutsakis ET: **Equations and calculations for fermentations of butyric acid bacteria**. *Biotechnol Bioeng* 1984, **26**:174-187.

**
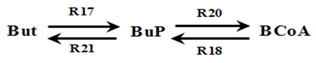
**

**Figure S1. The restructured butyrate formation - butyrate ressimilation of the ABE pathway*.*** The original lumped reactions R17 and R18 in Figure 1 are split and two new reactions are added. Here R17 is the reaction from butyrate to butyryl-phosphate, R18 is the reaction from butyryl-CoA to butyryl-phosphate, and R21 and R20 are the reverse reactions of R17 and R18, respectively.


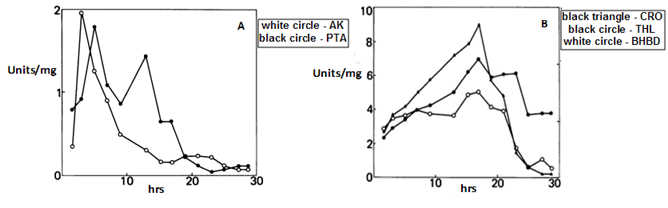


**Figure S2. The enzyme activity curves of AK, PTA, CRO, THL and BHBD.** The enzyme activity curves of AK (white circles) and PTA (black circles) are shown in A [5]. The enzyme activity curves of CRO (black triangle), THL (black circle) and BHBD (white circle) are shown in B [5]. The metric unit of the vertical axis in A and B is activity unit per mg; and the unit of the lateral axis is hr.


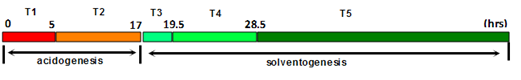


**Figure S3. The time division pattern (T)*.*** Time intervals are denoted by T1 – T5. The division is made according to the enzymes’ activity variations [3-7]. T1 (red) and T2 (orange) constitute acidogenesis; T3 (light green), T4 (green) and T5 (dark green) constitute solventogenesis. The boundary time points of T1 – T5 are drawn next to the intervals. And the lengths of T1 – T5 are proportional to the lengths of the time intervals they represent. The time here is model time, which is 10hr later than real time.

**
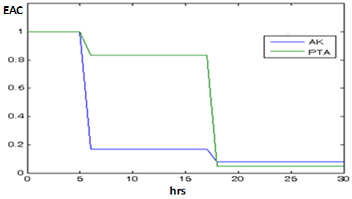
**

**Figure S4. The curves of the EAC functions of PTA and AK.** The curves of the EAC functions of PTA (green) and AK (blue) are shown. The EAC functions are approximated with 0^th^ splines. Each spline has the value that is equal to the ratio of the average activity level in an interval (defined in the time division pattern) to the maximum activity level. The time here is model time, which is 10hr later than real time.

**Table S1. Description of the overall time division T.** Computation result of the overall time division T according to the method described in Section S2. There are 5 intervals (T1 – T5) according to computation. Their specific boundary time points and corresponding biological features in ABE process are listed. The time here is model time, which is 10hr later than real time.

| Divided interval | Boundary time points of interval | Description of biological features |
| --- | --- | --- |
| T1 | (0, 5hr) | The vigorous period of acidogenesis. PTB is at its peak, while BK is at a relatively low level compared to its peak [5].CoAT is at a low activity level. Solventogenic enzymes (AADC, AAD, BDH) are of zero activities [3]. |
| T2 | (5hr, 17hr) | The ending period of acidogenesis, in which the onset of solventogenesis is included. PTB activity decreases, BK activity decreases slightly [5]. CoAT activity increases to a relatively high level and the activities of AADC, AAD, BDH begin to increase [3, 5]. |
| T3 | (17hr, 19.5hr) | Solventogenic period, which includes the acid reutilization. BK is at its peak, while PTB activity is under the detection level [5]. AAD, BDH are at a relatively high level. CoAT activity is still high. |
| T4 | (19.5hr, 28.5hr) | BK is still at its peak, PTB is of zero activity [5]. The activities of AAD, BDH, CoAT are relatively high. |
| T5 | (28.5hr, +∞)^*^ | Solvent production reaches stationary status, acidogenic enzymes are of zero activities, and solventogenic enzymatic activities are at high levels [3, 5, 6]. |

^*^ Since bacteria can not grow infinitely, in real time-course experiments of C. acetobutylicum, the ending time is 60hr [14, 16, 18] and our computation is also made in this time scale, with a calibration of 10hr. Here (28.5hr, +∞) just means “after 28.5hr, and up till the ending time”.
